# Association of plasma microRNA expression with age, genetic background and functional traits in dairy cattle

**DOI:** 10.1038/s41598-018-31099-w

**Published:** 2018-08-28

**Authors:** Jason Ioannidis, Enrique Sánchez-Molano, Androniki Psifidi, F. Xavier Donadeu, Georgios Banos

**Affiliations:** 1 SRUC, Animal and Veterinary Sciences, Easter Bush, EH25 9RG Midlothian, UK; 2 The Roslin Institute and Royal (Dick) School of Veterinary Studies, University of Edinburgh, Easter Bush, EH25 9RG Midlothian, UK; 3 Royal Veterinary College, University of London, Hertfordshire, AL9 7TA UK

## Abstract

A number of blood circulating microRNAs (miRNAs) are proven disease biomarkers and have been associated with ageing and longevity in multiple species. However, the role of circulating miRNAs in livestock species has not been fully studied. We hypothesise that plasma miRNA expression profiles are affected by age and genetic background, and associated with health and production traits in dairy cattle. Using PCR arrays, we assessed 306 plasma miRNAs for effects of age (calves vs mature cows) and genetic background (control vs select lines) in 18 animals. We identified miRNAs which were significantly affected by age (26 miRNAs) and genetic line (5 miRNAs). Using RT-qPCR in a larger cow population (n = 73) we successfully validated array data for 12 age-related miRNAs, one genetic line-related miRNA, and utilised expression data to associate their levels in circulation with functional traits in these animals. Plasma miRNA levels were associated with telomere length (ageing/longevity indicator), milk production and composition, milk somatic cell count (mastitis indicator), fertility, lameness, and blood metabolites linked with body energy balance and metabolic stress. In conclusion, circulating miRNAs could provide useful selection markers for dairy cows to help improve health, welfare and production performance.

## Introduction

MicroRNAs (miRNAs) are short non-coding RNAs which have been shown to regulate multiple animal body functions including reproduction, metabolism and immunity^1^. Some miRNAs show tissue-specific expression^2^ and can be simultaneously detected in blood, making them potential biomarkers for a range of conditions such as liver disease^3^, cardiovascular disease^4^ and cancer^5^. Moreover, miRNA expression in body tissues markedly changes with age, and distinct miRNA profiles have been associated with longevity in a wide range of species from *C. elegans* to humans^6–11^. In livestock, circulating miRNA expression profiles are altered in response to caloric restriction^12^, parasitic infection^13^, pregnancy^14^ and oestrus^15^. Interestingly, miRNA responses to caloric restriction have been shown to depend on the genetic background in some species, suggesting that genetics may significantly influence miRNA expression^12^. Caloric restriction is associated with longevity, and studies conducted in mice showed that circulating miRNAs which normally increase during ageing returned to normal levels after caloric reduction, implicating changes in miRNA levels in the metabolic effects of ageing^16^.

Dairy is one of the largest agricultural industries and comprises a large portion of the worldwide rural economy. Over several decades, genetic selection within dairy herds has resulted in an increase in annual milk production. However, higher milk production has also increased the caloric needs of dairy cows, leading to negative energy balance and metabolic stress. High-producing dairy cows suffer from hormonal imbalance and suppressed immune function, leading to increased incidence of mastitis (udder infection), uterine infections and lameness, all of which intensify with cow age^17^. In addition to implied animal health and welfare issues, high incidence of disease and infertility lead to increased veterinary costs and involuntary culling, which increase the cost of maintaining healthy dairy herds^18,19^ and compromise the competitiveness of the sector.

During the last few decades, more comprehensive genetic selection strategies have been developed and implemented in order to improve reproductive performance and health while maintaining high milk production in dairy cattle^20,21^. It is now thought that a long-standing decline in dairy cow fertility has been halted and is beginning to be reversed as a result of such selection programmes and better nutritional management^20,22^. Importantly, the effects of genetic selection are cumulative and their benefits are expected to become increasingly more apparent in the following years.

Following up on previous research described above, we are specifically interested in exploring the potential application of circulating miRNAs as selection markers for overall genetic merit and performance, and more specifically for ageing, fertility and welfare traits in dairy cattle. In the present study, we hypothesise that circulating miRNA expression profiles in cows will be altered by age and genetic background. In addition, we hypothesise that some of these miRNAs may also be associated with important health, fertility, longevity and production traits. To our knowledge, this is the first reported exploratory analysis of circulating miRNA expression levels in the context of age, genetic background and production traits in farm animals.

## Results and Discussion

### Identification of age- and genetic background-associated miRNA candidates

**Figure 1:**
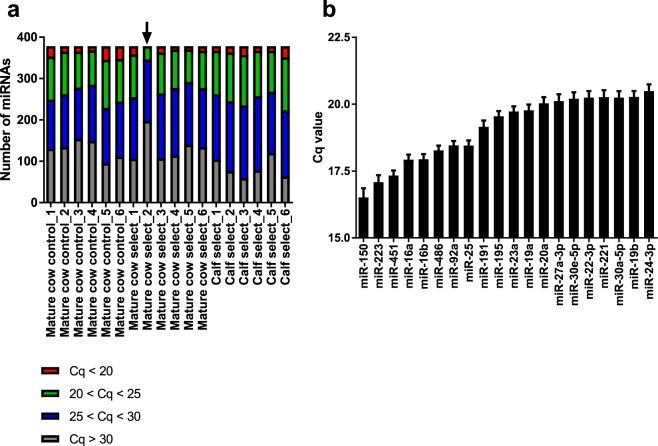
Plasma miRNA abundance. MiRNA detection analysis (**a**) and the expression of the 20 most abundant miRNAs in bovine plasma (**b**) based on a PCR array experiment. In (**b**), means ± SEM are shown.

In order to identify differences in circulating miRNAs between age groups and genetic lines of animals, we used PCR arrays to perform a screen of 378 plasma miRNAs in specific individual samples from mature cows (control and select lines) and calves (select line; see genetic line description in Methods). MiRNA abundance was similarly distributed across animal groups, with the exception of one sample in which all miRNAs had reduced detection (Fig. 1a, indicated by black arrow). This sample was excluded from further analyses. On average, 18 miRNAs were detected with Cq < 20, 103 with 20 < Cq < 25, 151 with 25 < Cq < 30, and 106 with Cq > 30. Many of the most abundant sequences found across samples (Fig. 1b) correspond to miRNAs that are enriched in blood cells (miR-451, 16a, 16b, 486)^23^. However, several miRNAs which according to our previous study^23^ are primarily expressed in plasma compared to blood cells, were also present (miR-195, 27a-3p, 22-3p, 30a-5p).

**Figure 2:**
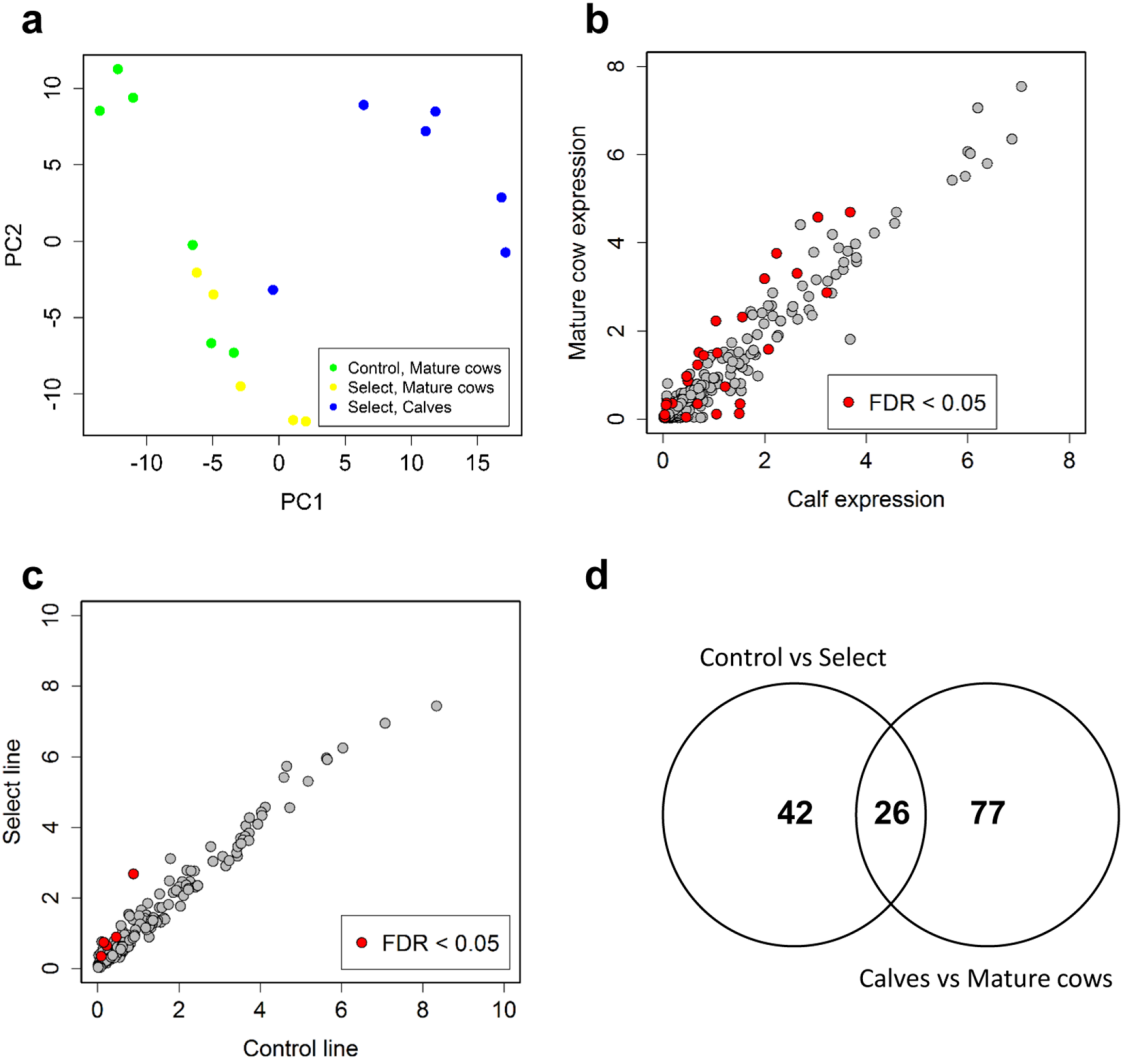
Results of differential miRNA expression analysis. (**a**) PCA plot of all samples used in the PCR array analysis. (**b**) Scatterplot showing the normalised expression level of all miRNAs in control vs select lines. (**c**) Scatterplot showing the normalised expression level of all miRNAs in mature cows vs calves from the select line. (**d**) Venn diagram showing the numbers of significantly different miRNAs across comparisons (P < 0.05) before correction for multiple testing.

Principal component analysis revealed a clear separation of samples between mature cows and calves from the select line but not between select and control mature cows (Fig. 2a). The expression of 26 miRNAs differed significantly between calves and mature cows (Supplementary Table S1, Fig. 2b), with 20 miRNAs changing by more than two-fold between groups. The largest-fold difference was observed for miR-127 (21-fold higher in calves). In addition, five miRNAs (miR-382, 323, 345-5p, 224 and 328) were differentially expressed in plasma samples between control and select mature cows following multiple testing correction (Supplementary Table S2, Fig. 2c). All five miRNAs had higher expression in select than control animals.

We used the NormFinder algorithm^24^ in array data to identify miRNAs that could be used as normalisers in subsequent RT-qPCR analysis. The miRNA with the most stable expression in plasma across all experimental groups was miR-101, followed closely by miR-148a (Supplementary Table S3). Taking into consideration both the stability values and miRNA abundance, we selected miR-148a and miR-19a as normalisers for RT-qPCR data.

In order to obtain insight into the functional relevance of the identified miRNA candidates, we determined the predicted biological pathways targeted by differentially expressed miRNAs (P < 0.05) associated with either age or genetic line (Supplementary Tables S1 and S2). Several tissue growth, metabolic and reproductive process pathways were overrepresented by predicted targets of miRNAs in each of the two group categories, including cell cycle, p53 signalling, insulin signalling, FOXO1 signalling, mTOR signalling, fatty acid metabolism, oocyte meiosis and steroid biosynthesis (P < 0.05, Supplementary File S1). A limitation of this approach is that miRNA function may not be conserved between human (where targeting interactions were first identified) and bovine.

### Validation and characterisation of differentially expressed miRNAs by RT-qPCR

Following our array screen, we selected 19 miRNAs for individual validation using RT-qPCR, considering their ranking in terms of abundance, fold-change and statistical significance in age and genetic line comparisons in the PCR array experiment. Changes in 12 out of 14 miRNAs that had varied with age according to PCR array were validated by RT-qPCR (Table 1). In contrast, changes in expression levels across genetic lines could be validated for only one of the five candidate miRNAs identified by PCR array (miR-15a, Table 1).

**Table 1:** Validation of PCR array results. Comparison of miRNA expression data obtained by PCR array and by RT-qPCR.

miRNA	Comparison	Array Fold-change	Array P-value	RT-qPCR Fold-change	RT-qPCR P-value
miR-126-5p	Age	2.05	0.029	2.18	0.001
miR-127	Age	0.05	0.042	0.08	<0.001
miR-140	Age	0.21	0.058	0.22	0.001
miR-192	Age	3.68	0.054	3.56	<0.001
miR-205	Age	5.35	0.025	12.90	<0.001
miR-23a	Age	2.11	0.025	1.83	0.157
miR-27a-3p	Age	3.15	0.026	2.50	<0.001
miR-29a	Age	3.38	0.025	2.80	0.015
miR-29c	Age	2.71	0.026	2.75	0.023
miR-380-3p	Age	0.08	0.048	0.08	<0.001
miR-31	Age	5.62	0.026	4.60	0.001
miR-215	Age	3.50	0.034	2.76	0.004
miR-154c	Age	0.07	0.042	0.04	<0.001
miR-378b	Age	2.52	0.034	1.40	0.337
miR-323	Line	5.99	0.038	2.16	0.108
miR-345-5p	Line	4.78	0.038	1.16	0.488
miR-328	Line	2.31	0.039	0.75	0.325
miR-15a	Line	1.91	0.080	1.44	0.038
miR-382	Line	6.48	0.038	Not detected	Not detected

Benjamini-Hochberg corrected p-values are shown under ‘Array P-value’ column. N = 4–11 biological replicates per group.

**Figure 3:**
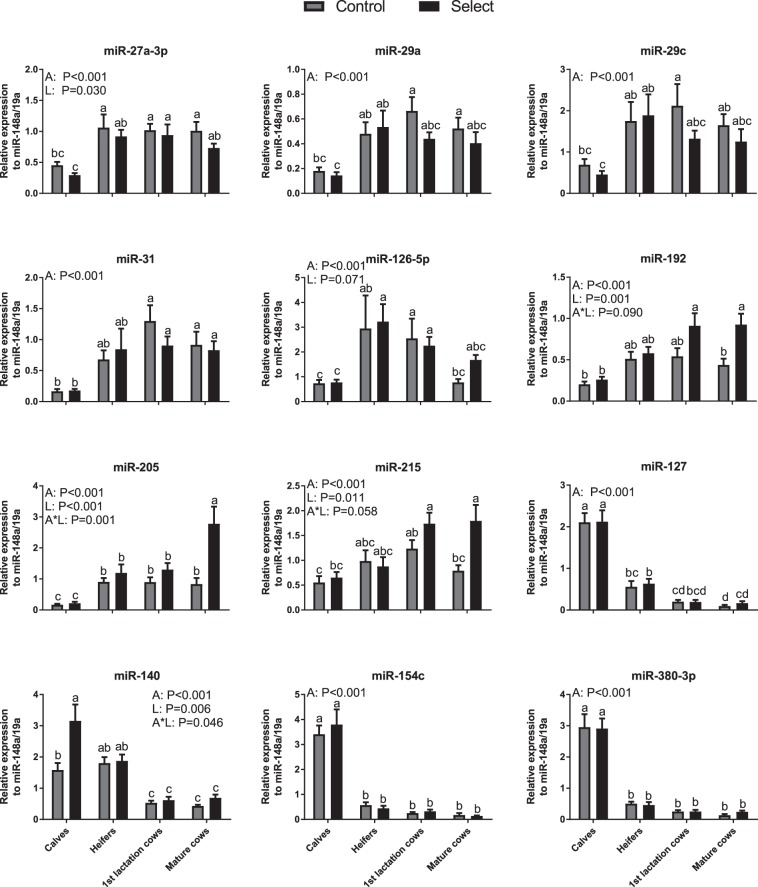
Expression profiles of validated miRNAs. Normalised expression profiles of miRNAs across age groups (horizontal axes) and genetic lines (control vs select). Data are shown as mean ± SEM. In each graph, p-values (<0.1) from two-way ANOVA analyses corresponding to main effects of age group (A), genetic line (L) and the interaction (A*L) are shown. In addition, means with different superscripts (abc) are different (P < 0.05). N = 6–12 animals per group.

Subsequent analyses were focused on the 12 validated age-related miRNAs. According to our previous data^23^, nearly all of these miRNAs are expressed at higher levels in plasma samples than blood cells. Changes in circulating levels of some of the miRNAs were consistent with previous studies in other species, including miR-126-5p^25,26^, miR-127^16,27^, miR-154c^27^ and miR-29 family miRNAs^7^. Moreover, profiling all 12 miRNAs by RT-qPCR across a wider age range, including calves, heifers, 1^st^ lactation and mature milking cows of each of control and select lines, revealed that these miRNAs could be divided in two different groups based on their expression pattern in plasma (Fig. 3). MiRNAs in the first group markedly increased in levels between calves and heifers, followed either by sustained high levels in 1^st^ lactation and mature cows (e.g., miR-27a-3p, miR-29a, miR-29c, miR-31) or a divergence in levels between the two lines resulting in higher miRNA expression in the select line at the mature cow stage (e.g., miR-192, miR-205, miR-215). In contrast, another group of miRNAs abruptly decreased in levels between calves (heifers in the case of miR-140) and later age groups, with lowest plasma levels occurring in 1^st^ lactation and mature cows (e.g., miR-127, miR-140, miR-154c and miR-380-3p).

Thus, a key finding in our study was that the most pronounced changes in circulating miRNA levels occurred early in life, namely between the calves and heifers groups. This finding is consistent with results from humans at different stages of life, from pre-natal to late adulthood, showing most pronounced changes for many circulating miRNAs occurring between children and young adult stages^28^. Considering the generic roles of miRNAs in regulation of tissue growth and differentiation, and, for many of them, their developmental stage-specific patterns of expression, profound changes in circulating miRNAs in association with rapid body tissue growth and maturation early during life are not unexpected. Indeed, an increase in the levels of miR-29a and other members of the same family in different tissues during post-natal growth has been shown to target growth-promoting genes thus contributing to eventual body growth cessation in mice^29^. Consistent with this, miR-29a levels markedly increased between calves and heifers in our study. Another miRNA identified in our study, miR-140, is known to actively regulate growth plate development and therefore bone length^30^, again highlighting the functional relevance of the identified changes in circulating miRNAs for calf growth. Moreover, in chicken, a group of seven circulating miRNAs were identified as potential predictive biomarkers of puberty onset^31^. Interestingly, those included two of the miRNAs upregulated with age in our study, miR-29a and miR-215. In light of these findings, the miRNAs identified in our study could provide useful biomarkers of important growth and development traits in cattle, and future studies should fully elucidate the role of these miRNAs during post-natal development.

Interestingly, levels of three of the 12 miRNAs analysed (miR-192, miR-205, miR-215) progressively increased in select relative to control cows across age categories, being significantly higher in select cows at the mature stage (Fig. 3). One of these miRNAs, miR-205, is expressed in the mammary gland of ruminants and its expression varies during the lactation cycle^32,33^. In addition, all three miRNAs are associated with liver metabolism or liver disease in a range of species. MiR-205 is responsible for the deregulation of lipid metabolism in hepatoma cells through targeting acyl-CoA synthase^34^ and miR-215 is a biomarker of hepatocellular carcinoma^35^. MiR-192 is among the most abundant miRNAs in the mammalian liver^36–38^ and it is differentially expressed in mouse models of non-alcoholic fatty liver disease^38^ and drug-induced liver injury^39^. An important finding is that the magnitude of change in miR-192 levels in non-alcoholic fatty liver disease depends on the genetic background (i.e. strain) of the mice used^38^. The importance of genetic background in response to stressful stimuli such as caloric restriction has also been previously highlighted in a chicken study^12^. Finally, miR-192 serum levels increase with age and also under caloric restriction conditions in mice^16^. Thus, the detected differences in plasma miRNA levels between genetic lines in this study could reflect differential tissue responses (presumably involving the liver and/or mammary gland) imposed by metabolic stress in high-production (select) cows, differences that likely become amplified in later life (i.e. in mature cows) due to the accumulating effects of recurrent lactations and ageing.

### Association of miRNA levels with animal functional traits

Correlation analyses showed a strong negative association of several miRNAs with telomere length, which is an indicator of animal ageing and longevity^40^ (Table 2; Supplementary Fig. S1). Multiple other studies have found an association between telomere length and several types of cancer in humans^17,41,42^. In addition, a study by Okada *et al*.^43^ revealed a significant up-regulation of miR-140 in late vs early passage mesenchymal stem cells and Uziel *et al*.^44^ showed down-regulation of this miRNA in breast cancer cells, thus indicating a potential anti-tumoral function. Similarly, miR-154c and miR-380-3p are down-regulated in several types of human cancers^45–48^, thus also indicating the potential tumour-suppressing function of these microRNAs.

**Table 2:** Significant correlations between animal traits and individual miRNAs.

Animal trait	miRNA	R	SE
Telomere length (ageing indicator)	miR-140	−0.64	0.15
Telomere length (ageing indicator)	miR-154c	−0.58	0.17
Telomere length (ageing indicator)	miR-380-3p	−0.82	0.07
Milk yield	miR-192	0.48	0.15
Milk yield	miR-323	0.69	0.11
Milk yield	miR-380-3p	0.88	0.04
Milk protein yield	miR-192	0.48	0.15
Milk protein yield	miR-323	0.68	0.12
Milk protein yield	miR-380-3p	0.90	0.04
Milk fat yield	miR-140	0.65	0.14
Milk fat yield	miR-154c	0.68	0.12
Milk fat yield	miR-323	0.59	0.15
Milk fat yield	miR-380-3p	0.92	0.03
Milk fat yield	miR-345-5p	−0.70	0.20
Milk somatic cell count (mastitis indicator)	miR-127	0.61	0.14
Milk somatic cell count (mastitis indicator)	miR-140	0.60	0.16
Milk somatic cell count (mastitis indicator)	miR-154c	0.85	0.06
Milk somatic cell count (mastitis indicator)	miR-380-3p	0.76	0.09
Fertility	miR-345-5p	0.92	0.05
Lameness	miR-127	−0.76	0.09
Lameness	miR-380-3p	0.74	0.10
Beta-Hydroxybutyrate	miR-205	0.50	0.10
Non Esterified Fatty Acids	miR-323	0.42	0.11

Significant threshold for p-values was computed through a Bonferroni correction considering 4 independent groups of miRNA and 5 independent groups of animal traits. R = correlation coefficient; SE = standard error.

There were significant correlations of miRNA profiles with milk production traits (yield, and protein and fat content) which were mainly positive (Table 2; Supplementary Fig. S1), suggesting a role in enhancing metabolic processes associated with lactation. Thus, miR-140 is known to be up-regulated in the liver of dairy cattle during negative energy balance, an altered metabolic state that occurs in early postpartum due to the high energy demand to support lactation^49^. In addition, miR-192 is expressed in bovine mammary gland^32^, and its changing levels in plasma could be a reflection of tissue changes due to the aforementioned caloric expenditure associated to the increase in milk production. MiR-154c is involved in lipid and protein metabolism^50^, therefore indicating its possible role in the mobilization of body fat and protein to support lactation. Interestingly, comparative mapping in other ruminant species linked the expression of miR-154c and miR-380 to a bovine QTL already identified for milk production and fat content^51^.

Most of the microRNAs found to be associated with health traits (mastitis, fertility, lameness) (Table 2; Supplementary Fig. S1) are known to be linked to the immune response and inflammation. For example, miR-127 is up-regulated in senescent fibroblasts and its expression changes during bone destruction and other inflammatory processes^52,53^, thus potentially explaining its association with lameness and mastitis. MiR-140 has been already associated with milk somatic cell count (a mastitis indicator) in other species through comparative mapping with bovine QTL^51^. Future work could investigate whether these miRNAs have potential use as early life selection biomarkers for production performance, longevity and welfare.

In summary, in the present study, we identified plasma circulating miRNAs whose expression is affected by age and genetic selection in dairy cattle. To our knowledge, this is the first exploratory study of circulating miRNAs in the context of ageing in dairy cattle. Our findings show that major changes in circulating miRNAs occur early during post-natal life in cattle suggesting important roles during body growth and maturation. In addition, we identify significant associations between a subset of these miRNAs and important animal traits associated with longevity, health and production. These miRNAs may prove to be useful selection and/or predictive biomarkers for traits of interest to the dairy industry. Future work should determine the functional implications of age- and genetic line-related differences in circulating miRNA levels in cattle to maximise their potential for selection breeding programmes aimed at improving animal productivity.

## Methods

### Ethics approval

The scientific work presented in this manuscript was approved by the Animal Experimentation Committee (AEC) of Scotland’s Rural College (Ref. ED AE 22-2016). This approval ensures compliance with Animal (Scientific Procedures) Act 1986. The experiment was contacted under Home Office regulations (Home Office Project Licence Number: PPL 60/4278).

### Study design

Blood samples were collected from a total of 73 animals at Crichton Royal Farm, located in Scotland, UK. This herd consisted of control and select genetic lines, as part of an ongoing long-term selection experiment^54^. Cows of the select line were daughters of top sires available internationally regarding their genetic merit for total milk content (fat and protein yield), whereas cows of the control line were daughters of sires with average genetic merit for these traits^54^. Select sires were bred into the select line starting in 1973 and control sires were bred into the control line starting in 1976. Each year, four to five control and select bulls were selected based on their predicted transmitting abilities and randomly mated with cows of the respective genetic line. All animals in the present study were purely of the Holstein-Friesian breed. More details regarding the control and select genetic lines are in Veerkamp *et al*.^54^. Furthermore, all animals were also equally divided into two feeding groups based on high and low fibre diets, as part of a long-standing feeding experiment^54^.

The sampled animals in the present study belonged to four different age groups: female calves of average age 7.5 days, heifers around 1 year old, 1^st^ lactation cows approximately 2 years old and mature cows approximately 4 years of age. Thirty-six control and 37 select animals were sampled from the two genetic lines. Animal sampling information is summarised in Supplementary Table S4.

### Sample collection and processing

Blood (10 mL) was collected in BD Vacutainer K2E collection tubes (Becton Dickinson, USA) from the tail vein using 18 G veterinary needles (Becton Dickinson). Two independent blood samples were drawn from the same animal each time and the sample with the least visual haemolysis was used in the study. Blood samples were kept at 4 °C and centrifuged at 1,900 × g for 10 min at 4 °C in a Jouan BR4i centrifuge (Thermo Electron, USA) to separate cells from plasma. Three quarters of the supernatant (approximately 3 mL) were aspirated and centrifuged again at 16,000 × g for 10 min at 4 °C to pellet residual cells and debris. The top three quarters of the supernatant (approximately 2 mL) were aspirated and passed through a 1.2 μm Minisart syringe filter (Sartorius, Germany) to remove any remaining cells and debris^55^. After filtering, samples were split in two 600 μL aliquots and frozen at −80 °C until further use.

### RNA extraction

RNA was extracted from 200 μL of plasma using the miRNeasy plasma kit (Qiagen), following the manufacturer’s instructions. As part of the standard protocol, each sample was spiked-in with 5.6 × 10^8^ copies of synthetic cel-miR-39-3p (Qiagen), which was used for quality control purposes in the PCR array experiment described below. RNA was eluted to a final volume of 12 μL in RNase-free water.

### PCR array profiling

Samples from control mature cows (n = 6), select mature cows (n = 6) and select calves (n = 6) were analysed using custom-made 384-well miScript PCR arrays (Qiagen). Our previous studies identified a total of 378 mature miRNAs in bovine plasma^15,56^ which were assayed in the custom arrays following pre-amplification using the miScript PreAMP kit (Qiagen) as per the manufacturer’s instructions. Before array analyses, RNA (2 μL) was reverse transcribed using the miScript II RT kit (Qiagen) following the manufacturer’s instructions. MiRNA expression levels were normalised using the global mean procedure^57^. Statistical analysis involved t-test comparisons between a) mature cows and calves of the select genetic line and b) control and select mature cows. Only miRNAs with Cq < 30 in 3 or more samples from at least one of the two groups in each comparison were included in the analysis, thus respectively resulting in 306 and 269 miRNAs in each of the above comparisons. All analyses were conducted on log-transformed data [log_2_(x + 1)] and results with FDR < 0.05 were considered as statistically significant. All graphs were generated using GraphPad Prism 5 or the R programming language core package^58^.

Some useful miRNA differences might have been missed in this stage of the study because of the small number of animals included. In addition, our approach would not allow the discovery of novel miRNAs, since the list of miRNAs to be measured was pre-defined. However, our existing miRNA data from previous studies^15,23,56^ ensured the majority of the detectable miRNAs in bovine plasma, which might provide insights into function or be used as biomarkers, were profiled in this study.

### RT-qPCR validation

RNA (2 μL) was reverse transcribed in 10 μL reactions using the miScript II RT kit (Qiagen) following the manufacturer’s instructions. MiRNAs with low abundance (on average Cq > 23, but assessed on a gene-by-gene basis) were pre-amplified in a 25 μL reaction using the miScript PreAMP kit (Qiagen) following the manufacturer’s instructions. QPCR was run using the miScript SYBR Green PCR Kit (Qiagen) on a Stratagene MX3000P qPCR System (Agilent, USA). A fluorescence threshold of 0.02 was used to determine Cq values. Gene expression data were exported from the MxPro software into Microsoft Excel and Minitab 17, for processing and statistical analysis, respectively. Statistical analysis included a two-way ANOVA using Tukey’s pairwise comparison test on transformed data. Outliers were detected manually and they were further confirmed using Grubb’s outlier test. All graphs were prepared using GraphPad Prism 5.

### Pathway enrichment analysis

Pathway enrichment analysis was carried out using the online tool miRPath 3.0 (accessed 13/07/2018) with default settings^59^. An independent miRPath query was generated for two sets of miRNAs identified in the comparisons outlined above. Experimentally validated targets for the selected miRNAs were identified using Tarbase 7.0 through the miRPath interface. Pathways involved in neoplasia and other diseases were removed from analyses. A potential limitation of our approach is the prediction of bovine miRNA function using target interactions that originate in human studies. However, this was prompted by the lack of miRNA target information in the cow.

### Associations with phenotypic traits

Milk yield and content (fat, protein) are routinely measured on each individual animal in the farm of study and recorded daily. Milk somatic cell count, an indicator of mastitis, is also recorded at the same time. Lactation milk yield, content and milk somatic cell count are calculated from these daily records using the methodology proposed by the International Committee for Animal Recording^60^. All animals are daily monitored on the farm by qualified personnel and any disease incidence is addressed and recorded by a veterinarian. All these data are included in the farm database which is being updated systematically. In addition, the telomere length at birth had been previously measured on 662 animals equally distributed across genetic lines and feeding groups based on blood samples collected in 2009–2014, using a qPCR protocol described in Seeker *et al*.^61^. Telomere length at birth is considered a biomarker predictor of animal ageing and longevity^40^.

For the purposes of the present study, phenotypic records of the 73 animals with microRNA profiles included: telomere length at birth, milk yield and content in the lactation of sampling (1st lactation and mature cow age groups only), and health related traits including milk somatic cell count (1st lactation and mature cow age groups only) and number of disease episodes pertaining to clinical mastitis, lameness and reproductive problems. Additionally, plasma levels of beta-hydroxybutyrate (BHB) and non-esterified fatty acids (NEFA), shown to be related with body energy balance, peri-parturient diseases and metabolic stress^62^, were quantified using an IL 650 Biochemisty Analyser (Werfen, UK).

A univariate model was fitted first for each phenotypic trait and miRNAs in order to identify significant fixed effects using Wald tests. Fixed effects included genetic line, age group, feeding group, month of sampling, age and month of calving (when applicable) and) lactation number (when applicable). To estimate the association between phenotypic traits and individual microRNA profiles, bivariate analyses were performed including the fixed effects from the univariate model. Principal component analyses of animal phenotypic traits and miRNA profiles were performed to determine the number of independent tests based on the distinct variation patterns derived. In total, four independent miRNA and five independent trait tests were defined. A Bonferroni correction for multiple testing was then applied to the significance level for the correlation results.

## Electronic supplementary material

Supplementary Information

## Data Availability

The datasets generated during and/or analysed during the current study are available from the corresponding author on reasonable request.
