# Supplementary material for: Association of plasma microRNA expression with age, genetic background and functional traits in dairy cattle
Source: Sci Rep. 2018 Aug 28;8:12955. doi: 10.1038/s41598-018-31099-w (PMC6113302; doi:10.1038/s41598-018-31099-w)
Supplement: Supplementary file 1 — Supplementary Information [file 41598_2018_31099_MOESM1_ESM.pdf]

Supplementary Information

Association of plasma microRNA expression with age, genetic background and functional traits in dairy cattle

Jason Ioannidis, Enrique Sánchez-Molano, Androniki Psifidi, F. Xavier Donadeu and Georgios Banos

Supplementary Figure S1

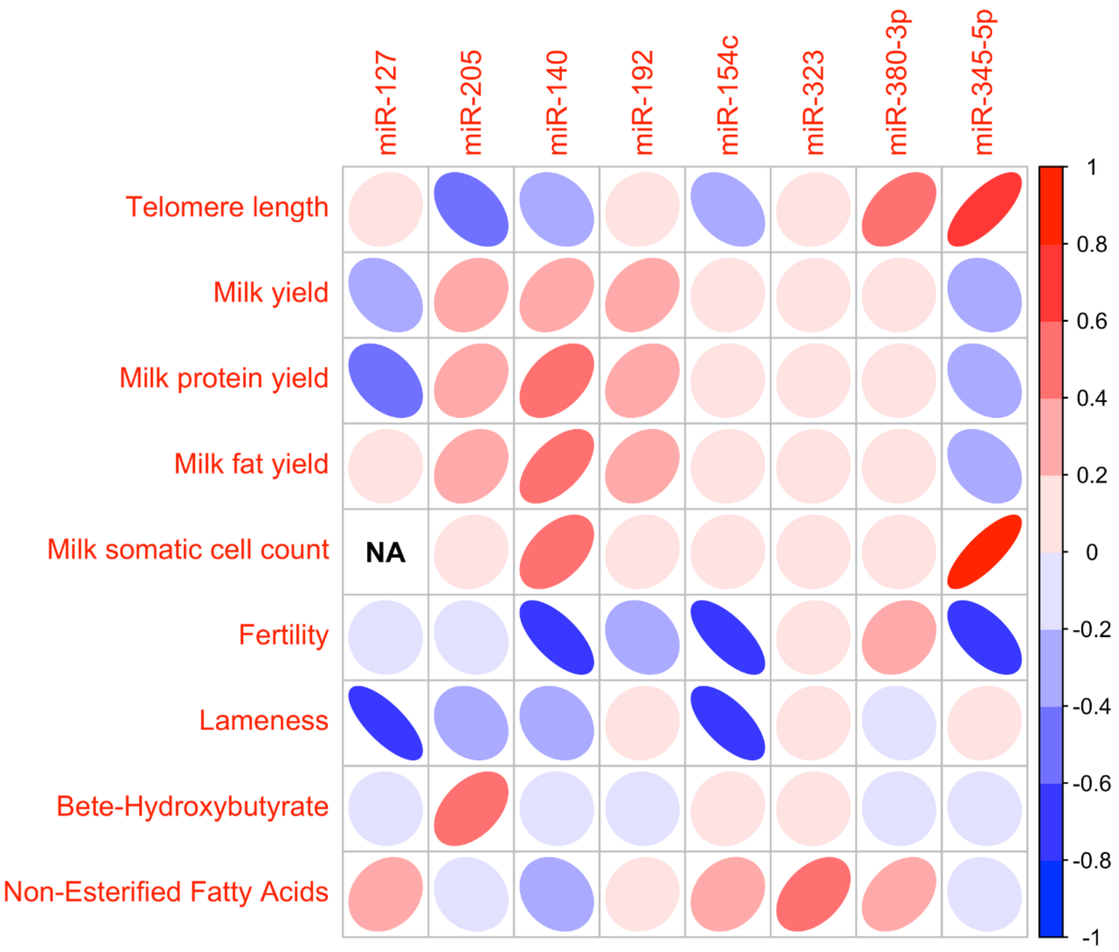

Supplementary Figure S1. Correlations between traits and individual microRNAs. Only microRNAs that were significant for a trait have been considered. Correlation between miR-127 and milk somatic cell count (NA) could not be estimated. Strength of correlation is indicated both by colour intensity and shape.

Supplementary Tables

Supplementary Table S1. MiRNA differences between calves and mature cows. MiRNAs with differential expression in plasma between select calves and mature cows, using PCR arrays. Benjamini-Hochberg corrected p-values are shown under ‘P-value’.

MiRNA	Calf - normalised expression	Mature cow - normalised expression	Fold-change (Cow / Calf)	P-value
miR-31	0.04	0.25	5.62	0.026
miR-205	0.05	0.29	5.35	0.025
miR-215	1.05	3.68	3.50	0.034
miR-29a	3.70	12.52	3.38	0.025
miR-27a-3p	7.27	22.92	3.15	0.026
miR-362-3p	0.02	0.07	3.06	0.042
miR-143	0.63	1.85	2.94	0.019
miR-29c	2.99	8.11	2.71	0.026
miR-378b	0.38	0.96	2.52	0.034
miR-23b-3p	0.74	1.72	2.31	0.026
miR-29d-3p	0.60	1.34	2.25	0.026
miR-132	0.13	0.28	2.16	0.014
miR-23a	11.82	24.90	2.11	0.025
miR-660	0.40	0.82	2.05	0.026
miR-126-5p	1.95	3.98	2.05	0.029
miR-142-5p	5.21	8.87	1.70	0.014
miR-30c	1.09	1.83	1.67	0.026
miR-378c	0.02	0.03	1.58	0.029
miR-423-3p	8.33	6.32	0.76	0.042
miR-484	3.21	2.00	0.62	0.044
miR-1307	1.33	0.67	0.51	0.014
miR-6123	0.60	0.27	0.45	0.026
miR-483	1.86	0.27	0.15	0.043
miR-380-3p	1.07	0.08	0.08	0.048
miR-154c	0.37	0.03	0.07	0.042
miR-127	1.82	0.09	0.05	0.042

Supplementary Table S2. MiRNA differences between genetic lines. MiRNAs with significantly different expression levels between control and select mature milking cows using PCR arrays. Benjamini-Hochberg corrected p-values are shown under 'P-value'.

MiRNA	Control -normalised expression	Select - normalised expression	Fold-change (Select / Control)	P-value
miR-382	0.84	5.42	6.48	0.038
miR-323	0.11	0.67	5.99	0.038
miR-345-5p	0.06	0.27	4.78	0.038
miR-224	0.17	0.56	3.36	0.038
miR-328	0.37	0.85	2.31	0.039

Supplementary Table S3. MiRNA stability in bovine plasma. The ten miRNAs with the most stable expression across all samples in the PCR array dataset, sorted by ascending stability value generated using the NormFinder algorithm.

MiRNA	NormFinder stability value	Cq-value
bta-miR-101	0.199	24.5
bta-miR-148a	0.201	22.9
bta-miR-532	0.219	27.0
bta-miR-19a	0.232	19.7
bta-miR-19b	0.241	20.2
bta-miR-15a	0.247	22.0
bta-miR-16a	0.250	17.9
bta-miR-20a	0.251	19.9
bta-miR-30e-5p	0.252	20.1
bta-miR-16b	0.255	17.9

Supplementary Table S4. Details of experimental design.

Age group, genetic line	No. of animals	Age (days, median)	Standard Deviation
Calves, control line	9	7	4.2
Calves, select line	12	8	6.8
Heifers, control line	7	384	31.7
Heifers, select line	6	370	15.7
1st lactation milking cows, control line	11	771	36.9
1st lactation milking cows, select line	8	773	52.7
Mature milking cows* control line	9	1,462	418.3
Mature milking cows*, select line	11	1,472	282.0

*Included cows at 2nd (n=8), 3rd (n=10), 4th (n=1) and 5th (n=1) lactations

Supplementary File S1

Pathways targetted by miRNAs which were differentially expressed between calves and mature cows (P < 0.05)

KEGG pathway	Group	P-value	No. genes affected	No. of miRNAs targetting pathway	MiRNAs targetting pathway																																					
Hippo signaling pathway	Signalling: development, organ size, apoptosis	0.000	100	35	miR-362-3p	miR-199a-3p	miR-149-5p	miR-140-5p	miR-27a-3p	miR-31-5p	miR-214-5p	miR-181b-5p	miR-432-5p	miR-205-5p	miR-192-5p	miR-143-5p	miR-199b-5p	miR-199a-5p	miR-29a-5p	miR-455-5p	miR-126-5p	miR-200a-5p	miR-331-3p	miR-127-5p	miR-23b-3p	miR-493-5p	miR-146a-5p	miR-424-3p	miR-100-5p	miR-485-5p	miR-455-3p	miR-299-5p	miR-450a-5p	miR-377-5p	miR-483-5p	miR-379-5p	miR-660-5p	miR-296-3p	miR-23a-5p			
Cell cycle	Cell cycle	0.000	88	35	miR-27a-3p	miR-181b-5p	miR-199a-5p	miR-29a-5p	miR-146a-5p	miR-199b-5p	miR-296-3p	miR-149-5p	miR-450a-5p	miR-455-5p	miR-126-5p	miR-23b-3p	miR-205-5p	miR-192-5p	miR-100-5p	miR-199a-3p	miR-485-5p	miR-299-5p	miR-331-3p	miR-214-5p	miR-140-5p	miR-424-3p	miR-31-5p	miR-377-5p	miR-455-3p	miR-362-3p	miR-200a-5p	miR-432-5p	miR-660-5p	miR-379-5p	miR-132-5p	miR-127-5p	miR-29c-5p	miR-23a-5p	miR-369-3p			
TGF-beta signaling pathway	Signalling: cell cycle, apoptosis	0.000	57	35	miR-29a-5p	miR-205-5p	miR-100-5p	miR-192-5p	miR-27a-3p	miR-199a-3p	miR-199a-5p	miR-140-5p	miR-31-5p	miR-199b-5p	miR-296-3p	miR-377-5p	miR-432-5p	miR-23b-3p	miR-127-5p	miR-126-5p	miR-660-5p	miR-331-3p	miR-299-5p	miR-23a-5p	miR-149-5p	miR-455-5p	miR-379-5p	miR-485-5p	miR-424-3p	miR-455-3p	miR-132-5p	miR-181b-5p	miR-362-3p	miR-200a-5p	miR-143-5p	miR-450a-5p	miR-214-5p	miR-493-5p	miR-146a-5p			
Adherens junction	Cell: cell-cell adhesion, cell movement	0.000	56	33	miR-27a-3p	miR-126-5p	miR-379-5p	miR-362-3p	miR-31-5p	miR-377-5p	miR-140-5p	miR-432-5p	miR-455-3p	miR-199a-3p	miR-181b-5p	miR-192-5p	miR-331-3p	miR-450a-5p	miR-205-5p	miR-100-5p	miR-493-5p	miR-23b-3p	miR-29a-5p	miR-132-5p	miR-485-5p	miR-660-5p	miR-144-5p	miR-214-5p	miR-146a-5p	miR-296-3p	miR-149-5p	miR-199b-5p	miR-199a-5p	miR-23a-5p	miR-143-5p	miR-200a-5p	miR-455-5p					
Protein processing in endoplasmic reticulum	Gene expression	0.000	111	38	miR-23b-3p	miR-126-5p	miR-27a-3p	miR-199a-3p	miR-140-5p	miR-483-5p	miR-362-3p	miR-450a-5p	miR-181b-5p	miR-192-5p	miR-199a-5p	miR-214-5p	miR-31-5p	miR-29c-5p	miR-29a-5p	miR-100-5p	miR-299-5p	miR-146a-5p	miR-149-5p	miR-432-5p	miR-205-5p	miR-377-5p	miR-455-5p	miR-379-5p	miR-493-5p	miR-369-3p	miR-23a-5p	miR-144-5p	miR-296-3p	miR-331-3p	miR-143-5p	miR-660-5p	miR-455-3p	miR-199b-5p	miR-132-5p	miR-200a-5p	miR-127-5p	miR-424-3p
Oocyte meiosis	Reproduction	0.000	74	33	miR-27a-3p	miR-126-5p	miR-23b-3p	miR-29a-5p	miR-181b-5p	miR-149-5p	miR-369-3p	miR-199a-3p	miR-140-5p	miR-192-5p	miR-455-3p	miR-362-3p	miR-31-5p	miR-424-3p	miR-205-5p	miR-146a-5p	miR-331-3p	miR-100-5p	miR-296-3p	miR-432-5p	miR-199a-5p	miR-143-5p	miR-200a-5p	miR-455-5p	miR-377-5p	miR-660-5p	miR-29c-5p	miR-485-5p	miR-132-5p	miR-199b-5p	miR-214-5p	miR-450a-5p	miR-493-5p					
Ubiquitin mediated proteolysis	Gene expression	0.000	92	35	miR-23b-3p	miR-126-5p	miR-362-3p	miR-27a-3p	miR-331-3p	miR-296-3p	miR-181b-5p	miR-29a-5p	miR-200a-5p	miR-100-5p	miR-149-5p	miR-192-5p	miR-199a-3p	miR-140-5p	miR-432-5p	miR-205-5p	miR-660-5p	miR-424-3p	miR-214-5p	miR-377-5p	miR-132-5p	miR-146a-5p	miR-299-5p	miR-455-5p	miR-199b-5p	miR-199a-5p	miR-31-5p	miR-143-5p	miR-144-5p	miR-455-3p	miR-485-5p	miR-493-5p	miR-379-5p	miR-23a-5p	miR-450a-5p			
FoxO signaling pathway	Signalling: apoptosis, metabolism, cell cycle	0.000	90	35	miR-23b-3p	miR-214-5p	miR-362-3p	miR-455-3p	miR-126-5p	miR-199b-5p	miR-199a-5p	miR-140-5p	miR-29a-5p	miR-205-5p	miR-27a-3p	miR-296-3p	miR-127-5p	miR-31-5p	miR-192-5p	miR-181b-5p	miR-100-5p	miR-432-5p	miR-331-3p	miR-146a-5p	miR-199a-3p	miR-485-5p	miR-455-5p	miR-143-5p	miR-132-5p	miR-149-5p	miR-299-5p	miR-660-5p	miR-450a-5p	miR-424-3p	miR-200a-5p	miR-379-5p	miR-369-3p	miR-23a-5p	miR-377-5p			
ErbB signaling pathway	Signalling: survival, migration, proliferation, differentiation	0.000	58	32	miR-27a-3p	miR-31-5p	miR-200a-5p	miR-199b-5p	miR-199a-5p	miR-455-5p	miR-205-5p	miR-132-5p	miR-23b-3p	miR-140-5p	miR-29a-5p	miR-362-3p	miR-192-5p	miR-126-5p	miR-181b-5p	miR-485-5p	miR-143-5p	miR-146a-5p	miR-127-5p	miR-432-5p	miR-199a-3p	miR-660-5p	miR-450a-5p	miR-331-3p	miR-149-5p	miR-100-5p	miR-455-3p	miR-493-5p	miR-29c-5p	miR-296-3p	miR-299-5p	miR-424-3p						
Thyroid hormone signaling pathway	Signalling: Growth, development, metabolism	0.000	76	33	miR-27a-3p	miR-29a-5p	miR-205-5p	miR-126-5p	miR-100-5p	miR-23b-3p	miR-660-5p	miR-31-5p	miR-192-5p	miR-362-3p	miR-331-3p	miR-199a-3p	miR-127-5p	miR-200a-5p	miR-181b-5p	miR-149-5p	miR-485-5p	miR-379-5p	miR-146a-5p	miR-296-3p	miR-140-5p	miR-493-5p	miR-143-5p	miR-199b-5p	miR-199a-5p	miR-455-5p	miR-424-3p	miR-369-3p	miR-455-3p	miR-29c-5p	miR-450a-5p	miR-432-5p	miR-132-5p					
p53 signaling pathway	Signalling: cell cycle, apoptosis, DNA damage repair	0.000	50	33	miR-27a-3p	miR-140-5p	miR-331-3p	miR-181b-5p	miR-455-3p	miR-199a-3p	miR-23b-3p	miR-29a-5p	miR-100-5p	miR-200a-5p	miR-379-5p	miR-126-5p	miR-660-5p	miR-455-5p	miR-192-5p	miR-362-3p	miR-146a-5p	miR-296-3p	miR-424-3p	miR-199b-5p	miR-149-5p	miR-205-5p	miR-127-5p	miR-214-5p	miR-199a-5p	miR-493-5p	miR-132-5p	miR-31-5p	miR-485-5p	miR-299-5p	miR-143-5p	miR-377-5p	miR-483-5p					
Signaling pathways regulating pluripotency of stem cells	Signalling: pluripotency	0.000	86	35	miR-126-5p	miR-31-5p	miR-200a-5p	miR-485-5p	miR-27a-3p	miR-29a-5p	miR-146a-5p	miR-331-3p	miR-181b-5p	miR-140-5p	miR-424-3p	miR-23b-3p	miR-199a-3p	miR-192-5p	miR-205-5p	miR-432-5p	miR-143-5p	miR-362-3p	miR-199b-5p	miR-199a-5p	miR-144-5p	miR-455-5p	miR-100-5p	miR-29c-5p	miR-149-5p	miR-296-3p	miR-660-5p	miR-455-3p	miR-23a-5p	miR-214-5p	miR-450a-5p	miR-377-5p	miR-379-5p	miR-493-5p	miR-132-5p			
Lysine degradation	Metabolism	0.000	30	25	miR-29a-5p	miR-140-5p	miR-126-5p	miR-27a-3p	miR-660-5p	miR-23b-3p	miR-296-3p	miR-377-5p	miR-181b-5p	miR-485-5p	miR-331-3p	miR-149-5p	miR-192-5p	miR-455-5p	miR-362-3p	miR-31-5p	miR-200a-5p	miR-199a-3p	miR-199a-5p	miR-29c-5p	miR-205-5p	miR-493-5p	miR-146a-5p	miR-424-3p	miR-299-5p													
ECM-receptor interaction	Cell: cell adhesion, migration, differentiation, proliferation, apoptosis and mechanical force receptors	0.000	43	32	miR-27a-3p	miR-29a-5p	miR-199b-5p	miR-199a-3p	miR-377-5p	miR-485-5p	miR-199a-5p	miR-23a-5p	miR-31-5p	miR-299-5p	miR-23b-3p	miR-126-5p	miR-140-5p	miR-450a-5p	miR-362-3p	miR-493-5p	miR-432-5p	miR-331-3p	miR-455-3p	miR-149-5p	miR-181b-5p	miR-205-5p	miR-192-5p	miR-214-5p	miR-127-5p	miR-100-5p	miR-455-5p	miR-379-5p	miR-143-5p	miR-296-3p	miR-660-5p	miR-424-3p						
Focal adhesion	Cell: motility, survival, proliferation	0.000	126	36	miR-27a-3p	miR-214-5p	miR-192-5p	miR-331-3p	miR-377-5p	miR-149-5p	miR-199b-5p	miR-199a-3p	miR-296-3p	miR-199a-5p	miR-299-5p	miR-432-5p	miR-455-3p	miR-200a-5p	miR-181b-5p	miR-29a-5p	miR-23b-3p	miR-146a-5p	miR-205-5p	miR-424-3p	miR-126-5p	miR-127-5p	miR-31-5p	miR-362-3p	miR-455-5p	miR-140-5p	miR-450a-5p	miR-379-5p	miR-132-5p	miR-100-5p	miR-143-5p	miR-485-5p	miR-493-5p	miR-660-5p	miR-144-5p	miR-23a-5p		
Neurotrophin signaling pathway	Signalling: neuron differentiation and survival	0.000	78	32	miR-27a-3p	miR-100-5p	miR-296-3p	miR-23b-3p	miR-146a-5p	miR-192-5p	miR-331-3p	miR-126-5p	miR-205-5p	miR-29a-5p	miR-362-3p	miR-149-5p	miR-31-5p	miR-140-5p	miR-660-5p	miR-455-3p	miR-181b-5p	miR-132-5p	miR-200a-5p	miR-199b-5p	miR-199a-5p	miR-485-5p	miR-199a-3p	miR-29c-5p	miR-493-5p	miR-214-5p	miR-369-3p	miR-432-5p	miR-127-5p	miR-143-5p	miR-455-5p	miR-424-3p						
Endocytosis	Cell: endocytosis	0.000	124	36	miR-205-5p	miR-126-5p	miR-23b-3p	miR-27a-3p	miR-100-5p	miR-199b-5p	miR-199a-3p	miR-199a-5p	miR-181b-5p	miR-29a-5p	miR-192-5p	miR-485-5p	miR-331-3p	miR-127-5p	miR-140-5p	miR-455-5p	miR-362-3p	miR-660-5p	miR-379-5p	miR-149-5p	miR-432-5p	miR-31-5p	miR-296-3p	miR-214-5p	miR-450a-5p	miR-144-5p	miR-455-3p	miR-146a-5p	miR-377-5p	miR-493-5p	miR-132-5p	miR-23a-5p	miR-424-3p	miR-200a-5p	miR-143-5p	miR-29c-5p		
Wnt signaling pathway	Signalling: cell fate, progenitor proliferation, cell cycle	0.000	85	32	miR-362-3p	miR-27a-3p	miR-31-5p	miR-192-5p	miR-126-5p	miR-23b-3p	miR-140-5p	miR-146a-5p	miR-199a-5p	miR-29a-5p	miR-149-5p	miR-100-5p	miR-143-5p	miR-432-5p	miR-200a-5p	miR-424-3p	miR-455-5p	miR-660-5p	miR-199b-5p	miR-296-3p	miR-331-3p	miR-199a-3p	miR-181b-5p	miR-23a-5p	miR-205-5p	miR-379-5p	miR-127-5p	miR-485-5p	miR-455-3p	miR-493-5p	miR-132-5p	miR-214-5p						
mTOR signaling pathway	Signalling: metabolism, cytosketeton, survival	0.000	43	26	miR-27a-3p	miR-455-3p	miR-126-5p	miR-192-5p	miR-331-3p	miR-181b-5p	miR-149-5p	miR-450a-5p	miR-29a-5p	miR-199b-5p	miR-199a-5p	miR-23b-3p	miR-31-5p	miR-485-5p	miR-140-5p	miR-205-5p	miR-199a-3p	miR-455-5p	miR-143-5p	miR-100-5p	miR-146a-5p	miR-424-3p	miR-296-3p	miR-362-3p	miR-369-3p	miR-132-5p												
AMPK signaling pathway	Signalling: cellular energy regulation, metabolism	0.001	78	32	miR-23b-3p	miR-192-5p	miR-29a-5p	miR-100-5p	miR-493-5p	miR-27a-3p	miR-199a-3p	miR-485-5p	miR-455-3p	miR-296-3p	miR-331-3p	miR-181b-5p	miR-140-5p	miR-362-3p	miR-205-5p	miR-132-5p	miR-146a-5p	miR-126-5p	miR-29c-5p	miR-31-5p	miR-149-5p	miR-379-5p	miR-424-3p	miR-455-5p	miR-127-5p	miR-199b-5p	miR-199a-5p	miR-299-5p	miR-200a-5p	miR-432-5p	miR-143-5p	miR-450a-5p						
Spliceosome	Gene expression	0.001	77	37	miR-27a-3p	miR-146a-5p	miR-23b-3p	miR-432-5p	miR-149-5p	miR-181b-5p	miR-205-5p	miR-126-5p	miR-29a-5p	miR-200a-5p	miR-199b-5p	miR-199a-5p	miR-424-3p	miR-127-5p	miR-493-5p	miR-455-5p	miR-199a-3p	miR-100-5p	miR-362-3p	miR-31-5p	miR-450a-5p	miR-660-5p	miR-143-5p	miR-377-5p	miR-144-5p	miR-485-5p	miR-140-5p	miR-455-3p	miR-379-5p	miR-331-3p	miR-296-3p	miR-192-5p	miR-299-5p	miR-23a-5p	miR-132-5p	miR-369-3p	miR-29c-5p	
mRNA surveillance pathway	Gene expression	0.001	61	29	miR-23b-3p	miR-27a-3p	miR-29a-5p	miR-31-5p	miR-181b-5p	miR-331-3p	miR-424-3p	miR-455-3p	miR-192-5p	miR-199a-3p	miR-146a-5p	miR-140-5p	miR-450a-5p	miR-432-5p	miR-205-5p	miR-127-5p	miR-362-3p	miR-132-5p	miR-296-3p	miR-149-5p	miR-199b-5p	miR-199a-5p	miR-126-5p	miR-143-5p	miR-455-5p	miR-200a-5p	miR-379-5p	miR-660-5p	miR-100-5p									
TNF signaling pathway	Signalling: apoptosis, inflammation	0.001	69	31	miR-126-5p	miR-23b-3p	miR-362-3p	miR-31-5p	miR-192-5p	miR-27a-3p	miR-29a-5p	miR-146a-5p	miR-199a-3p	miR-485-5p	miR-455-5p	miR-143-5p	miR-181b-5p	miR-149-5p	miR-144-5p	miR-100-5p	miR-140-5p	miR-331-3p	miR-299-5p	miR-200a-5p	miR-379-5p	miR-199b-5p	miR-199a-5p	miR-493-5p	miR-23a-5p	miR-205-5p	miR-214-5p	miR-296-3p	miR-455-3p	miR-660-5p	miR-127-5p							
Circadian rhythm	Biological clock	0.002	23	21	miR-127-5p	miR-432-5p	miR-27a-3p	miR-126-5p	miR-362-3p	miR-23b-3p	miR-146a-5p	miR-450a-5p	miR-485-5p	miR-493-5p	miR-140-5p	miR-455-3p	miR-100-5p	miR-149-5p	miR-192-5p	miR-199a-3p	miR-181b-5p	miR-31-5p	miR-205-5p	miR-331-3p	miR-29a-5p																	
Regulation of actin cytoskeleton	Cell: cytoskeleton, motility	0.002	119	38	miR-23b-3p	miR-199a-3p	miR-27a-3p	miR-29a-5p	miR-192-5p	miR-126-5p	miR-493-5p	miR-181b-5p	miR-200a-5p	miR-149-5p	miR-214-5p	miR-146a-5p	miR-199b-5p	miR-199a-5p	miR-362-3p	miR-31-5p	miR-331-3p	miR-140-5p	miR-450a-5p	miR-127-5p	miR-143-5p	miR-100-5p	miR-485-5p	miR-455-5p	miR-296-3p	miR-299-5p	miR-432-5p	miR-455-3p	miR-369-3p	miR-377-5p	miR-132-5p	miR-205-5p	miR-660-5p	miR-144-5p	miR-29c-5p	miR-424-3p	miR-379-5p	miR-483-5p
Fatty acid biosynthesis	Metabolism	0.002	6	14	miR-192-5p	miR-149-5p	miR-23b-3p	miR-27a-3p	miR-100-5p	miR-199a-3p	miR-140-5p	miR-29a-5p	miR-331-3p	miR-377-5p	miR-455-5p	miR-181b-5p	miR-424-3p	miR-455-3p																								
HIF-1 signaling pathway	Signalling: Hypoxia, anaerobic metabolism	0.002	67	33	miR-126-5p	miR-27a-3p	miR-199a-3p	miR-485-5p	miR-455-5p	miR-23b-3p	miR-362-3p	miR-192-5p	miR-143-5p	miR-146a-5p	miR-296-3p	miR-29a-5p	miR-31-5p	miR-140-5p	miR-132-5p	miR-205-5p	miR-100-5p	miR-181b-5p	miR-127-5p	miR-331-3p	miR-149-5p	miR-432-5p	miR-483-5p	miR-379-5p	miR-299-5p	miR-455-3p	miR-29c-5p	miR-199b-5p	miR-199a-5p	miR-23a-5p	miR-214-5p	miR-660-5p	miR-450a-5p					
Sphingolipid signaling pathway	Signalling: stress response, apoptosis	0.002	71	29	miR-181b-5p	miR-126-5p	miR-23b-3p	miR-27a-3p	miR-331-3p	miR-199a-3p	miR-127-5p	miR-146a-5p	miR-132-5p	miR-199b-5p	miR-199a-5p	miR-140-5p	miR-424-3p	miR-455-3p	miR-362-3p	miR-31-5p	miR-192-5p	miR-29a-5p	miR-205-5p	miR-450a-5p	miR-100-5p	miR-200a-5p	miR-485-5p	miR-455-5p	miR-660-5p	miR-149-5p	miR-143-5p	miR-432-5p	miR-493-5p									
RNA transport	Gene expression	0.004	97	34	miR-27a-3p	miR-181b-5p	miR-126-5p	miR-29a-5p	miR-205-5p	miR-192-5p	miR-149-5p	miR-140-5p	miR-362-3p	miR-146a-5p	miR-199b-5p	miR-199a-3p	miR-199a-5p	miR-23b-3p	miR-424-3p	miR-29c-5p	miR-132-5p	miR-432-5p	miR-493-5p	miR-127-5p	miR-377-5p	miR-455-3p	miR-144-5p	miR-660-5p	miR-331-3p	miR-100-5p	miR-214-5p	miR-450a-5p	miR-200a-5p	miR-299-5p	miR-296-3p	miR-143-5p	miR-379-5p	miR-31-5p				
N-Glycan biosynthesis	Metabolism	0.005	30	27	miR-23b-3p	miR-377-5p	miR-181b-5p	miR-455-5p	miR-493-5p	miR-369-3p	miR-192-5p	miR-31-5p	miR-432-5p	miR-205-5p	miR-100-5p	miR-132-5p	miR-450a-5p	miR-27a-3p	miR-126-5p	miR-362-3p	miR-660-5p	miR-299-5p	miR-424-3p	miR-29a-5p	miR-149-5p	miR-200a-5p	miR-140-5p	miR-199b-5p	miR-199a-5p	miR-199a-3p	miR-214-5p											
Progesterone-mediated oocyte maturation	Reproduction	0.006	55	34	miR-29a-5p	miR-23b-3p	miR-146a-5p	miR-331-3p	miR-27a-3p	miR-379-5p	miR-424-3p	miR-126-5p	miR-127-5p	miR-493-5p	miR-450a-5p	miR-149-5p	miR-143-5p	miR-199a-3p	miR-485-5p	miR-455-5p	miR-660-5p	miR-362-3p	miR-31-5p	miR-100-5p	miR-455-3p	miR-432-5p	miR-132-5p	miR-199b-5p	miR-199a-5p	miR-140-5p	miR-192-5p	miR-299-5p	miR-29c-5p	miR-181b-5p	miR-205-5p	miR-296-3p	miR-377-5p	miR-214-5p				
Estrogen signaling pathway	Endocrine	0.006	59	33	miR-23b-3p	miR-192-5p	miR-331-3p	miR-660-5p	miR-205-5p	miR-31-5p	miR-149-5p	miR-181b-5p	miR-455-3p	miR-362-3p	miR-199a-3p	miR-369-3p	miR-432-5p	miR-143-5p	miR-29a-5p	miR-199b-5p	miR-199a-5p	miR-27a-3p	miR-140-5p	miR-127-5p	miR-493-5p	miR-450a-5p	miR-126-5p	miR-485-5p	miR-455-5p	miR-146a-5p	miR-100-5p	miR-424-3p	miR-379-5p	miR-377-5p	miR-299-5p	miR-214-5p	miR-132-5p					
Inositol phosphate metabolism	Metabolism	0.012	35	22	miR-29a-5p	miR-126-5p	miR-23b-3p	miR-27a-3p	miR-192-5p	miR-181b-5p	miR-483-5p	miR-199a-3p	miR-455-5p	miR-100-5p	miR-455-3p	miR-205-5p	miR-424-3p	miR-331-3p	miR-146a-5p	miR-144-5p	miR-299-5p	miR-140-5p	miR-29c-5p	miR-132-5p	miR-149-5p	miR-31-5p																
Lysosome	Cellular processing	0.017	66	36	miR-23b-3p	miR-146a-5p	miR-27a-3p	miR-362-3p	miR-140-5p	miR-29a-5p	miR-214-5p	miR-199b-5p	miR-199a-5p	miR-299-5p	miR-455-3p	miR-331-3p	miR-432-5p	miR-199a-3p	miR-455-5p	miR-192-5p	miR-377-5p	miR-379-5p	miR-450a-5p	miR-143-5p	miR-181b-5p	miR-205-5p	miR-296-3p	miR-200a-5p	miR-126-5p	miR-660-5p	miR-483-5p	miR-100-5p	miR-127-5p	miR-31-5p	miR-369-3p	miR-485-5p	miR-493-5p	miR-149-5p	miR-132-5p	miR-424-3p		
Phosphatidylinositol signaling system	Signalling: environmental signal processing	0.019	46	31	miR-23b-3p	miR-192-5p	miR-199a-3p	miR-181b-5p	miR-205-5p	miR-424-3p	miR-455-3p	miR-29c-5p	miR-126-5p	miR-27a-3p	miR-485-5p	miR-455-5p	miR-143-5p	miR-331-3p	miR-132-5p	miR-199b-5p	miR-199a-5p	miR-100-5p	miR-146a-5p	miR-144-5p	miR-362-3p	miR-296-3p	miR-149-5p	miR-140-5p	miR-660-5p	miR-31-5p	miR-29a-5p	miR-369-3p	miR-432-5p	miR-483-5p	miR-127-5p							
Insulin signaling pathway	Metabolism	0.026	80	30	miR-126-5p	miR-181b-5p	miR-205-5p	miR-331-3p	miR-23b-3p	miR-27a-3p	miR-146a-5p	miR-29a-5p	miR-149-5p	miR-132-5p	miR-362-3p	miR-100-5p	miR-483-5p	miR-192-5p	miR-140-5p	miR-31-5p	miR-450a-5p	miR-143-5p	miR-660-5p	miR-199a-3p	miR-369-3p	miR-432-5p	miR-199b-5p	miR-199a-5p	miR-485-5p	miR-455-5p	miR-127-5p	miR-299-5p	miR-455-3p	miR-424-3p								
Valine, leucine and isoleucine degradation	Metabolism	0.042	24	14	miR-27a-3p	miR-140-5p	miR-205-5p	miR-23b-3p	miR-362-3p	miR-660-5p	miR-192-5p	miR-181b-5p	miR-493-5p	miR-29a-5p	miR-199b-5p	miR-199a-5p	miR-126-5p	miR-199a-3p																								
RNA degradation	Gene expression	0.044	47	34	miR-132-5p	miR-362-3p	miR-126-5p	miR-27a-3p	miR-146a-5p	miR-331-3p	miR-199a-3p	miR-149-5p	miR-140-5p	miR-192-5p	miR-181b-5p	miR-23b-3p	miR-29a-5p	miR-100-5p	miR-29c-5p	miR-31-5p	miR-143-5p	miR-199b-5p	miR-199a-5p	miR-214-5p	miR-296-3p	miR-660-5p	miR-424-3p	miR-200a-5p	miR-455-3p	miR-432-5p	miR-127-5p	miR-379-5p	miR-450a-5p	miR-23a-5p	miR-485-5p	miR-205-5p	miR-493-5p	miR-144-5p				

Pathways targetted by miRNAs which were differentially expressed between control and select genetic lines (P < 0.05)

KEGG pathway	Group	P-value	No. genes affected	No. of miRNAs targetting pathway	MiRNAs targetting pathway																	
Adherens junction	Cell: cell-cell adhesion, cell movement	0.000	46	16	miR-199a-5p	miR-138-5p	miR-214-5p	miR-224-5p	miR-146a-5p	miR-345-5p	miR-99b-5p	miR-187-5p	miR-497-5p	miR-196a-5p	miR-149-5p	miR-23b-5p	miR-323a-5p	miR-1224-5p	miR-380-5p	miR-486-5p	miR-486-5p		
Hippo signaling pathway	DNA damage repair	0.000	69	16	miR-497-5p	miR-224-5p	miR-146a-5p	miR-196a-5p	miR-214-5p	miR-138-5p	miR-424-3p	miR-1224-5p	miR-187-5p	miR-486-5p	miR-199a-5p	miR-149-5p	miR-345-5p	miR-154-5p	miR-23b-5p	miR-382-5p			
Lysine degradation	Metabolism	0.000	26	13	miR-199a-5p	miR-138-5p	miR-224-5p	miR-146a-5p	miR-196a-5p	miR-497-5p	miR-486-5p	miR-23b-5p	miR-149-5p	miR-187-5p	miR-345-5p	miR-1224-5p	miR-424-3p						
Protein processing in endoplasmic reticulum	Gene expression	0.000	87	19	miR-224-5p	miR-149-5p	miR-146a-5p	miR-497-5p	miR-138-5p	miR-345-5p	miR-214-5p	miR-424-3p	miR-196a-5p	miR-382-5p	miR-1224-5p	miR-187-5p	miR-486-5p	miR-23b-5p	miR-199a-5p	miR-154-5p	miR-323a-5p	miR-380-5p	miR-99b-5p
ECM-receptor interaction	Cell cycle	0.000	35	16	miR-138-5p	miR-1224-5p	miR-196a-5p	miR-486-5p	miR-380-5p	miR-23b-5p	miR-497-5p	miR-199a-5p	miR-382-5p	miR-224-5p	miR-154-5p	miR-214-5p	miR-424-3p	miR-149-5p	miR-323a-5p	miR-187-5p			
FoxO signaling pathway	Immunity	0.000	69	17	miR-424-3p	miR-196a-5p	miR-224-5p	miR-382-5p	miR-486-5p	miR-1224-5p	miR-497-5p	miR-199a-5p	miR-138-5p	miR-149-5p	miR-323a-5p	miR-23b-5p	miR-146a-5p	miR-345-5p	miR-99b-5p	miR-187-5p	miR-214-5p		
Endocytosis	Cell cycle	0.000	96	18	miR-1224-5p	miR-323a-5p	miR-224-5p	miR-23b-5p	miR-99b-5p	miR-187-5p	miR-196a-5p	miR-199a-5p	miR-345-5p	miR-486-5p	miR-154-5p	miR-146a-5p	miR-497-5p	miR-149-5p	miR-424-3p	miR-382-5p	miR-138-5p	miR-214-5p	
TGF-beta signaling pathway	Gene expression	0.000	38	17	miR-382-5p	miR-149-5p	miR-486-5p	miR-23b-5p	miR-224-5p	miR-196a-5p	miR-214-5p	miR-199a-5p	miR-497-5p	miR-424-3p	miR-345-5p	miR-99b-5p	miR-138-5p	miR-1224-5p	miR-323a-5p	miR-146a-5p	miR-187-5p		
Focal adhesion	Immunity	0.000	97	19	miR-224-5p	miR-23b-5p	miR-497-5p	miR-196a-5p	miR-199a-5p	miR-99b-5p	miR-323a-5p	miR-345-5p	miR-424-3p	miR-149-5p	miR-146a-5p	miR-138-5p	miR-214-5p	miR-187-5p	miR-382-5p	miR-380-5p	miR-154-5p	miR-486-5p	miR-1224-5p
Spliceosome	Gene expression	0.000	61	15	miR-224-5p	miR-486-5p	miR-99b-5p	miR-23b-5p	miR-497-5p	miR-138-5p	miR-199a-5p	miR-196a-5p	miR-149-5p	miR-345-5p	miR-146a-5p	miR-382-5p	miR-1224-5p	miR-323a-5p	miR-424-3p				
p53 signaling pathway	Gene expression	0.001	36	15	miR-345-5p	miR-149-5p	miR-486-5p	miR-497-5p	miR-424-3p	miR-196a-5p	miR-382-5p	miR-224-5p	miR-146a-5p	miR-23b-5p	miR-214-5p	miR-199a-5p	miR-138-5p	miR-187-5p	miR-1224-5p				
mTOR signaling pathway	Gene expression	0.001	33	16	miR-224-5p	miR-497-5p	miR-149-5p	miR-138-5p	miR-345-5p	miR-146a-5p	miR-382-5p	miR-196a-5p	miR-199a-5p	miR-99b-5p	miR-1224-5p	miR-154-5p	miR-486-5p	miR-23b-5p	miR-424-3p	miR-323a-5p			
Neurotrophin signaling pathway	Gene expression	0.002	57	16	miR-146a-5p	miR-382-5p	miR-1224-5p	miR-224-5p	miR-424-3p	miR-497-5p	miR-138-5p	miR-149-5p	miR-486-5p	miR-323a-5p	miR-196a-5p	miR-199a-5p	miR-23b-5p	miR-345-5p	miR-214-5p	miR-99b-5p			
Cell cycle	Cell cycle	0.002	59	17	miR-149-5p	miR-23b-5p	miR-146a-5p	miR-196a-5p	miR-497-5p	miR-1224-5p	miR-199a-5p	miR-99b-5p	miR-138-5p	miR-224-5p	miR-486-5p	miR-424-3p	miR-214-5p	miR-345-5p	miR-323a-5p	miR-187-5p	miR-382-5p		
RNA transport	Gene expression	0.003	74	19	miR-154-5p	miR-497-5p	miR-196a-5p	miR-382-5p	miR-138-5p	miR-224-5p	miR-380-5p	miR-149-5p	miR-23b-5p	miR-214-5p	miR-345-5p	miR-146a-5p	miR-323a-5p	miR-486-5p	miR-1224-5p	miR-424-3p	miR-199a-5p	miR-99b-5p	miR-187-5p
Steroid biosynthesis	Gene expression	0.005	10	8	miR-345-5p	miR-23b-5p	miR-497-5p	miR-224-5p	miR-99b-5p	miR-199a-5p	miR-424-3p	miR-196a-5p											
RNA degradation	Gene expression	0.006	40	16	miR-149-5p	miR-138-5p	miR-497-5p	miR-196a-5p	miR-380-5p	miR-214-5p	miR-23b-5p	miR-424-3p	miR-199a-5p	miR-224-5p	miR-382-5p	miR-146a-5p	miR-99b-5p	miR-345-5p	miR-1224-5p	miR-486-5p			
PI3K-Akt signaling pathway	Gene expression	0.009	133	19	miR-224-5p	miR-138-5p	miR-486-5p	miR-382-5p	miR-196a-5p	miR-146a-5p	miR-154-5p	miR-323a-5p	miR-23b-5p	miR-497-5p	miR-187-5p	miR-380-5p	miR-199a-5p	miR-149-5p	miR-214-5p	miR-99b-5p	miR-1224-5p	miR-345-5p	miR-424-3p
Oocyte meiosis	Gene expression	0.009	46	17	miR-224-5p	miR-196a-5p	miR-138-5p	miR-23b-5p	miR-345-5p	miR-149-5p	miR-424-3p	miR-99b-5p	miR-497-5p	miR-1224-5p	miR-146a-5p	miR-486-5p	miR-199a-5p	miR-187-5p	miR-323a-5p	miR-214-5p	miR-154-5p		
Sphingolipid signaling pathway	Gene expression	0.014	50	17	miR-382-5p	miR-424-3p	miR-486-5p	miR-196a-5p	miR-23b-5p	miR-1224-5p	miR-224-5p	miR-323a-5p	miR-345-5p	miR-497-5p	miR-154-5p	miR-99b-5p	miR-138-5p	miR-146a-5p	miR-187-5p	miR-199a-5p	miR-149-5p		
Gap junction	DNA damage repair	0.015	37	15	miR-224-5p	miR-146a-5p	miR-99b-5p	miR-23b-5p	miR-345-5p	miR-149-5p	miR-196a-5p	miR-486-5p	miR-187-5p	miR-323a-5p	miR-1224-5p	miR-424-3p	miR-497-5p	miR-199a-5p	miR-138-5p				
TNF signaling pathway	Gene expression	0.018	47	16	miR-323a-5p	miR-345-5p	miR-196a-5p	miR-497-5p	miR-149-5p	miR-224-5p	miR-382-5p	miR-138-5p	miR-154-5p	miR-146a-5p	miR-23b-5p	miR-199a-5p	miR-1224-5p	miR-486-5p	miR-214-5p	miR-99b-5p			
Insulin signaling pathway	Metabolism	0.019	60	16	miR-224-5p	miR-138-5p	miR-497-5p	miR-196a-5p	miR-486-5p	miR-154-5p	miR-149-5p	miR-23b-5p	miR-382-5p	miR-146a-5p	miR-1224-5p	miR-345-5p	miR-323a-5p	miR-199a-5p	miR-424-3p	miR-99b-5p			
Ubiquitin mediated proteolysis	Gene expression	0.023	60	19	miR-196a-5p	miR-497-5p	miR-23b-5p	miR-149-5p	miR-154-5p	miR-187-5p	miR-146a-5p	miR-486-5p	miR-424-3p	miR-345-5p	miR-382-5p	miR-214-5p	miR-224-5p	miR-138-5p	miR-1224-5p	miR-199a-5p	miR-380-5p	miR-323a-5p	miR-99b-5p
Dorso-ventral axis formation	Cell cycle	0.042	15	12	miR-149-5p	miR-497-5p	miR-199a-5p	miR-224-5p	miR-187-5p	miR-146a-5p	miR-154-5p	miR-196a-5p	miR-323a-5p	miR-23b-5p	miR-138-5p	miR-345-5p							
Regulation of actin cytoskeleton	Gene expression	0.047	80	19	miR-23b-5p	miR-199a-5p	miR-99b-5p	miR-497-5p	miR-138-5p	miR-196a-5p	miR-382-5p	miR-224-5p	miR-486-5p	miR-214-5p	miR-380-5p	miR-146a-5p	miR-149-5p	miR-187-5p	miR-323a-5p	miR-345-5p	miR-1224-5p	miR-154-5p	miR-424-3p
